# Carbamo­yl(di­amino­methyl­idene)aza­nium 3-nitro-5-oxo-4,5-di­hydro-1*H*-1,2,4-triazol-4-ide

**DOI:** 10.1107/S1600536813015699

**Published:** 2013-06-12

**Authors:** Xin-Ping Huang, Bo-Zhou Wang, Dong-Ping Li, Seik Weng Ng

**Affiliations:** a Xi’an Modern Chemistry Research Institute, Xi’an 710065, People’s Republic of China; b Department of Mathematics, Jining Teachers College, Wulanchabu 012000, People’s Republic of China; c Department of Chemistry, University of Malaya, 50603 Kuala Lumpur, Malaysia; d Chemistry Department, King Abdulaziz University, PO Box 80203 Jeddah, Saudi Arabia

## Abstract

In the anion of the title salt, C_2_H_7_N_4_O^+^·C_2_HN_4_O_3_^−^, the negative charge resides formally on the N^3^ atom of the triazole ring. In the crystal, the N^3^ and exocyclic O atoms are hydrogen-bond acceptors with respect to the formally double-bond iminium and amido N atoms of the cation. The cation and anion are almost planar (r.m.s. deviations = 0.012 and 0.051 Å, respectively), but they are slightly bent with respect to each other [dihedral angle = 12.6 (1)°]. In the crystal, adjacent anions and cations are linked by extensive N—H⋯N and N—H⋯O hydrogen bonds, generating a ribbon running along the *b*-axis direction.

## Related literature

For background to applications of similar compounds as propellants and explosives, see: Liu *et al.* (2006 ▶); Östmark *et al.* (2002 ▶). 
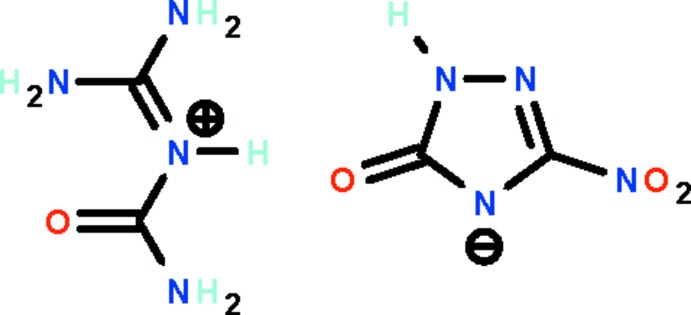


## Experimental

### 

#### Crystal data

C_2_H_7_N_4_O^+^·C_2_HN_4_O_3_^−^
*M* *_r_* = 232.18
Monoclinic, 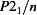

*a* = 3.7100 (5) Å
*b* = 13.4195 (19) Å
*c* = 18.033 (3) Å
β = 94.143 (3)°
*V* = 895.5 (2) Å^3^
*Z* = 4
Mo *K*α radiation
μ = 0.15 mm^−1^
*T* = 293 K
0.30 × 0.30 × 0.20 mm

#### Data collection

Bruker SMART APEX diffractometer
5217 measured reflections
2032 independent reflections
1297 reflections with *I* > 2σ(*I*)
*R*_int_ = 0.035

#### Refinement

*R*[*F* ^2^ > 2σ(*F* ^2^)] = 0.044
*wR*(*F* ^2^) = 0.116
*S* = 1.00
2032 reflections
177 parameters
All H-atom parameters refined
Δρ_max_ = 0.17 e Å^−3^
Δρ_min_ = −0.23 e Å^−3^

### 

Data collection: *APEX2* (Bruker, 2007 ▶); cell refinement: *SAINT* (Bruker, 2007 ▶); data reduction: *SAINT*; program(s) used to solve structure: *SHELXS97* (Sheldrick, 2008 ▶); program(s) used to refine structure: *SHELXL97* (Sheldrick, 2008 ▶); molecular graphics: *X-SEED* (Barbour, 2001 ▶); software used to prepare material for publication: *publCIF* (Westrip, 2010 ▶).

## Supplementary Material

Crystal structure: contains datablock(s) global, I. DOI: 10.1107/S1600536813015699/xu5702sup1.cif

Structure factors: contains datablock(s) I. DOI: 10.1107/S1600536813015699/xu5702Isup2.hkl

Supplementary material file. DOI: 10.1107/S1600536813015699/xu5702Isup3.cml

Additional supplementary materials:  crystallographic information; 3D view; checkCIF report

## Figures and Tables

**Table 1:** Hydrogen-bond geometry (Å, °)

*D*—H⋯*A*	*D*—H	H⋯*A*	*D*⋯*A*	*D*—H⋯*A*
N1—H1⋯O4^i^	0.87 (2)	1.97 (2)	2.819 (2)	166 (2)
N5—H2⋯N3	0.94 (2)	1.99 (3)	2.926 (3)	173 (2)
N5—H3⋯O1^ii^	0.90 (3)	2.13 (3)	3.005 (2)	164 (2)
N6—H4⋯O1	0.89 (2)	1.96 (2)	2.824 (2)	163 (2)
N8—H5⋯O1	0.95 (3)	2.15 (3)	2.966 (3)	142 (2)
N8—H6⋯O3^iii^	0.87 (2)	2.32 (3)	3.183 (3)	173 (2)
N7—H7⋯N2^iii^	0.90 (2)	2.03 (3)	2.913 (2)	166 (2)
N7—H8⋯O4	0.85 (2)	2.02 (2)	2.645 (2)	129 (2)

Symmetry codes: (i) 
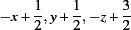
; (ii) 
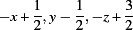
; (iii) 
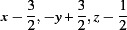
.

**Fig. 1.**
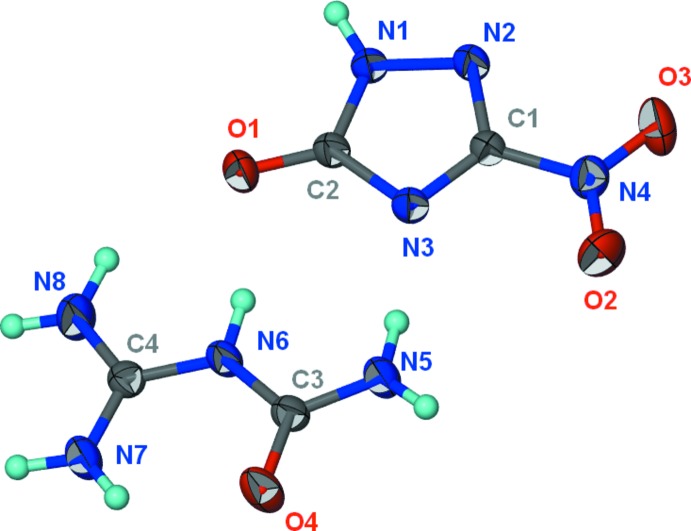
Thermal ellipsoid plot (Barbour, 2001) of C2H7N4O.C2HN4O3 at the 70% probability level; hydrogen atoms are drawn as spheres of arbitrary radius.

**Fig. 2.**
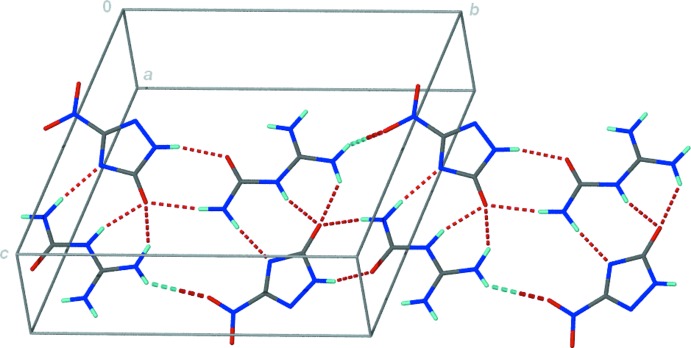
Packing diagram.

## supplementary crystallographic information

### Comment

We have reported organic compounds that do not possess carbon-bound hydrogen atoms; *N*-guanylurea dinitramide, NH_2_C(NH)NHC(O)NH_2_^.^NH(NO_2_)_2_ (Liu *et al.*, 2006), exemplifies such a compound that has been evaluated for use as a propellant and an insensitive-munitions explosive (Östmark *et al.*, 2002). The title salt (Scheme I, Fig. 1) features an (NH_2_)_2_C(NH)C(O)NH_2_ cation that has been protonated by 3-nitro-1,2,4-triazol-5-one, which is acidic owing to the electron-withdrawing nitro group. The N^3^ and exocyclic O atoms are hydrogen bond acceptors with respect to the formally double-bond iminium and amido N atoms of the cation. The cation and anion are planar but they are slightly bent with respect to each other. Adjacent ion-pairs are linked by extensive N···N and N···O hydrogen bonds to generate a ribbon structure (Table 1).

### Experimental

3-Nitro-1,2,4-triazol-5-one (26.0 g, 0.2 mol) was suspended in water (150 ml) kept at 303–313 K. Sodium hydroxide (8.2 g, 0.2 mol) dissolved in water (50 ml) was added. Guanylurea hydrochloride (27.8 g, 0.2 mol) dissolved in water (175 ml) was aded. The mixture was warmed to 323–333 K for 1.5 h. This was then cooled to 275–278 K. The solid material was collected and recrystallized from water (yield 35.0 g, 85% yield).

### Refinement

Hydrogen atoms were located in a difference Fourier map, and were freely refined.

### Crystal data

**Table:**

C_2_H_7_N_4_O^+^·C_2_HN_4_O_3_^−^	*F*(000) = 480
*M**_r_* = 232.18	*D*_x_ = 1.722 Mg m^−^^3^
Monoclinic, *P*2_1_/*n*	Mo *K*α radiation, λ = 0.71073 Å
Hall symbol: -P 2yn	Cell parameters from 976 reflections
*a* = 3.7100 (5) Å	θ = 2.3–24.8°
*b* = 13.4195 (19) Å	µ = 0.15 mm^−^^1^
*c* = 18.033 (3) Å	*T* = 293 K
β = 94.143 (3)°	Prism, yellow
*V* = 895.5 (2) Å^3^	0.30 × 0.30 × 0.20 mm
*Z* = 4	

### Data collection

**Table:**

Bruker SMART APEX diffractometer	1297 reflections with *I* > 2σ(*I*)
Radiation source: fine-focus sealed tube	*R*_int_ = 0.035
Graphite monochromator	θ_max_ = 27.5°, θ_min_ = 1.9°
ω scans	*h* = −4→4
5217 measured reflections	*k* = −14→17
2032 independent reflections	*l* = −22→21

### Refinement

**Table:**

Refinement on *F*^2^	Primary atom site location: structure-invariant direct methods
Least-squares matrix: full	Secondary atom site location: difference Fourier map
*R*[*F*^2^ > 2σ(*F*^2^)] = 0.044	Hydrogen site location: inferred from neighbouring sites
*wR*(*F*^2^) = 0.116	All H-atom parameters refined
*S* = 1.00	*w* = 1/[σ^2^(*F*_o_^2^) + (0.0564*P*)^2^] where *P* = (*F*_o_^2^ + 2*F*_c_^2^)/3
2032 reflections	(Δ/σ)_max_ = 0.001
177 parameters	Δρ_max_ = 0.17 e Å^−^^3^
0 restraints	Δρ_min_ = −0.23 e Å^−^^3^

### Fractional atomic coordinates and isotropic or equivalent isotropic displacement parameters (Å^2^)

**Table:**

	*x*	*y*	*z*	*U*_iso_*/*U*_eq_	
O1	0.3898 (4)	0.79686 (10)	0.74934 (8)	0.0387 (4)	
O2	0.9393 (5)	0.48678 (10)	0.86971 (9)	0.0526 (5)	
O3	1.2361 (4)	0.56912 (12)	0.95758 (9)	0.0502 (5)	
O4	0.0042 (5)	0.49420 (10)	0.59852 (8)	0.0429 (4)	
N1	0.7380 (5)	0.80376 (12)	0.86167 (9)	0.0309 (4)	
N2	0.9228 (5)	0.73874 (11)	0.90892 (8)	0.0301 (4)	
N3	0.6699 (5)	0.65784 (11)	0.80734 (9)	0.0288 (4)	
N4	1.0257 (5)	0.56440 (12)	0.90216 (9)	0.0343 (4)	
N5	0.3102 (5)	0.50638 (14)	0.71153 (9)	0.0356 (5)	
N6	0.1708 (5)	0.64808 (12)	0.64482 (9)	0.0304 (4)	
N7	−0.1373 (5)	0.66445 (15)	0.52843 (10)	0.0364 (5)	
N8	0.0601 (6)	0.80164 (14)	0.59459 (12)	0.0465 (6)	
C1	0.8697 (5)	0.65588 (13)	0.87193 (10)	0.0257 (4)	
C2	0.5814 (5)	0.75613 (14)	0.80084 (10)	0.0281 (5)	
C3	0.1543 (6)	0.54422 (14)	0.64972 (10)	0.0291 (5)	
C4	0.0251 (6)	0.70408 (14)	0.58795 (10)	0.0283 (4)	
H1	0.703 (6)	0.8651 (17)	0.8753 (12)	0.042 (6)*	
H2	0.434 (6)	0.5511 (18)	0.7446 (12)	0.046 (7)*	
H3	0.290 (7)	0.440 (2)	0.7190 (14)	0.064 (8)*	
H4	0.257 (7)	0.6841 (19)	0.6834 (13)	0.053 (7)*	
H5	0.177 (8)	0.8310 (19)	0.6379 (16)	0.076 (9)*	
H6	−0.034 (7)	0.8406 (18)	0.5601 (13)	0.052 (7)*	
H7	−0.248 (6)	0.7032 (18)	0.4929 (13)	0.051 (7)*	
H8	−0.162 (6)	0.6015 (18)	0.5248 (13)	0.046 (7)*	

### Atomic displacement parameters (Å^2^)

**Table:**

	*U*^11^	*U*^22^	*U*^33^	*U*^12^	*U*^13^	*U*^23^
O1	0.0502 (10)	0.0337 (8)	0.0302 (8)	0.0054 (7)	−0.0120 (7)	0.0002 (6)
O2	0.0776 (13)	0.0250 (8)	0.0544 (10)	0.0059 (8)	−0.0017 (9)	−0.0022 (7)
O3	0.0493 (10)	0.0527 (10)	0.0459 (10)	0.0033 (8)	−0.0145 (8)	0.0133 (8)
O4	0.0670 (11)	0.0287 (8)	0.0307 (8)	−0.0121 (7)	−0.0130 (7)	0.0012 (6)
N1	0.0418 (11)	0.0222 (8)	0.0274 (9)	0.0024 (8)	−0.0054 (8)	−0.0039 (7)
N2	0.0362 (11)	0.0268 (8)	0.0262 (9)	0.0003 (7)	−0.0050 (7)	−0.0025 (7)
N3	0.0338 (10)	0.0244 (9)	0.0272 (9)	0.0000 (7)	−0.0036 (7)	−0.0030 (7)
N4	0.0375 (11)	0.0305 (9)	0.0348 (10)	0.0012 (8)	0.0027 (8)	0.0045 (8)
N5	0.0521 (12)	0.0251 (9)	0.0276 (10)	−0.0028 (9)	−0.0099 (9)	0.0016 (8)
N6	0.0434 (11)	0.0234 (8)	0.0231 (9)	−0.0028 (8)	−0.0071 (8)	−0.0013 (7)
N7	0.0484 (12)	0.0306 (10)	0.0283 (10)	−0.0027 (9)	−0.0103 (9)	0.0033 (8)
N8	0.0717 (16)	0.0240 (10)	0.0413 (12)	0.0037 (10)	−0.0129 (11)	0.0019 (9)
C1	0.0271 (11)	0.0260 (10)	0.0238 (10)	0.0004 (8)	0.0004 (8)	−0.0009 (8)
C2	0.0341 (12)	0.0246 (10)	0.0252 (10)	−0.0009 (8)	−0.0014 (9)	−0.0020 (8)
C3	0.0362 (12)	0.0263 (10)	0.0244 (10)	−0.0025 (9)	−0.0002 (9)	−0.0013 (8)
C4	0.0313 (11)	0.0275 (10)	0.0263 (10)	−0.0003 (9)	0.0024 (8)	0.0003 (8)

### Geometric parameters (Å, º)

**Table:**

O1—C2	1.253 (2)	N5—H2	0.94 (2)
O2—N4	1.226 (2)	N5—H3	0.90 (3)
O3—N4	1.224 (2)	N6—C4	1.352 (2)
O4—C3	1.240 (2)	N6—C3	1.398 (2)
N1—C2	1.363 (2)	N6—H4	0.89 (2)
N1—N2	1.369 (2)	N7—C4	1.306 (3)
N1—H1	0.87 (2)	N7—H7	0.90 (2)
N2—C1	1.304 (2)	N7—H8	0.85 (2)
N3—C1	1.335 (2)	N8—C4	1.320 (3)
N3—C2	1.362 (2)	N8—H5	0.95 (3)
N4—C1	1.447 (2)	N8—H6	0.87 (2)
N5—C3	1.320 (2)		
			
C2—N1—N2	111.56 (15)	H7—N7—H8	119 (2)
C2—N1—H1	127.4 (15)	C4—N8—H5	121.4 (16)
N2—N1—H1	120.3 (15)	C4—N8—H6	120.0 (16)
C1—N2—N1	100.07 (14)	H5—N8—H6	118 (2)
C1—N3—C2	102.08 (15)	N2—C1—N3	118.90 (16)
O3—N4—O2	124.25 (18)	N2—C1—N4	119.28 (17)
O3—N4—C1	118.53 (17)	N3—C1—N4	121.82 (16)
O2—N4—C1	117.21 (17)	O1—C2—N3	127.31 (18)
C3—N5—H2	117.0 (14)	O1—C2—N1	125.30 (18)
C3—N5—H3	118.0 (17)	N3—C2—N1	107.39 (16)
H2—N5—H3	125 (2)	O4—C3—N5	124.49 (19)
C4—N6—C3	125.80 (17)	O4—C3—N6	120.77 (18)
C4—N6—H4	113.2 (16)	N5—C3—N6	114.74 (18)
C3—N6—H4	120.6 (16)	N7—C4—N8	121.0 (2)
C4—N7—H7	120.5 (15)	N7—C4—N6	122.19 (18)
C4—N7—H8	120.5 (16)	N8—C4—N6	116.81 (19)
			
C2—N1—N2—C1	1.2 (2)	C1—N3—C2—O1	−179.1 (2)
N1—N2—C1—N3	−1.0 (2)	C1—N3—C2—N1	0.4 (2)
N1—N2—C1—N4	179.47 (16)	N2—N1—C2—O1	178.41 (19)
C2—N3—C1—N2	0.4 (2)	N2—N1—C2—N3	−1.1 (2)
C2—N3—C1—N4	179.90 (17)	C4—N6—C3—O4	1.9 (3)
O3—N4—C1—N2	−7.9 (3)	C4—N6—C3—N5	−178.53 (19)
O2—N4—C1—N2	172.69 (18)	C3—N6—C4—N7	−3.2 (3)
O3—N4—C1—N3	172.61 (18)	C3—N6—C4—N8	178.0 (2)
O2—N4—C1—N3	−6.8 (3)		

### Hydrogen-bond geometry (Å, º)

**Table:**

*D*—H···*A*	*D*—H	H···*A*	*D*···*A*	*D*—H···*A*
N1—H1···O4^i^	0.87 (2)	1.97 (2)	2.819 (2)	166 (2)
N5—H2···N3	0.94 (2)	1.99 (3)	2.926 (3)	173 (2)
N5—H3···O1^ii^	0.90 (3)	2.13 (3)	3.005 (2)	164 (2)
N6—H4···O1	0.89 (2)	1.96 (2)	2.824 (2)	163 (2)
N8—H5···O1	0.95 (3)	2.15 (3)	2.966 (3)	142 (2)
N8—H6···O3^iii^	0.87 (2)	2.32 (3)	3.183 (3)	173 (2)
N7—H7···N2^iii^	0.90 (2)	2.03 (3)	2.913 (2)	166 (2)
N7—H8···O4	0.85 (2)	2.02 (2)	2.645 (2)	129 (2)

Symmetry codes: (i) −*x*+1/2, *y*+1/2, −*z*+3/2; (ii) −*x*+1/2, *y*−1/2, −*z*+3/2; (iii) *x*−3/2, −*y*+3/2, *z*−1/2.
